# The Effects of a Prophylactic Knee Brace and Two Neoprene Knee Sleeves on the Performance of Healthy Athletes: A Crossover Randomized Controlled Trial

**DOI:** 10.1371/journal.pone.0050110

**Published:** 2012-11-21

**Authors:** Niyousha Mortaza, Ismail Ebrahimi, Ali Ashraf Jamshidi, Vahid Abdollah, Mohammad Kamali, Wan Abu Bakar Wan Abas, Noor Azuan Abu Osman

**Affiliations:** 1 Department of Orthotics and Prosthetics, Faculty of Rehabilitation, Tehran University of Medical Sciences, Tehran, Iran; 2 Department of Biomedical Engineering, Faculty of Engineering, University of Malaya, Kuala Lumpur, Malaysia; 3 Department of Physical Therapy, Faculty of Rehabilitation, Tehran University of Medical Sciences, Tehran, Iran; 4 Rehabilitation Research Center, Faculty of Rehabilitation, Tehran University of Medical Sciences, Tehran, Iran; Universidad Europea de Madrid, Spain

## Abstract

Knee injury is one of the major problems in sports medicine, and the use of prophylactic knee braces is an attempt to reduce the occurrence and/or severity of injuries to the knee joint ligament(s) without inhibiting knee mobility. The aim of the present study was to examine the effect of one recently designed prophylactic knee brace and two neoprene knee sleeves upon performance of healthy athletes. Thirty-one healthy male athletes (age = 21.2±1.5) volunteered as participants to examine the effect of prophylactic knee brace/sleeves on performance using isokinetic and functional tests. All subjects were tested in four conditions in a random order: 1. nonbraced (control) 2. using a neoprene knee sleeve 3. using a knee sleeve with four bilateral metal supports and 4. using a prophylactic knee brace. The study design was a crossover, randomized, controlled trial. Subjects completed single leg vertical jump, cross-over hop, and the isokinetic knee flexion and extension (at 60, 180, 300°/sec). Data were collected from the above tests and analyzed for jump height, cross-over hop distance, peak torque to body weight ratio and average power, respectively. Comparisons of these variables in the four testing conditions revealed no statistically significant difference (p>0.05). The selected prophylactic brace/sleeves did not significantly inhibit athletic performance which might verify that their structure and design have caused no complication in the normal function of the knee joint. Moreover, it could be speculated that, if the brace or the sleeves had any limiting effect, our young healthy athletic subjects were well able to generate a mean peak torque large enough to overcome this possible restriction. Further studies are suggested to investigate the long term effect of these prophylactic knee brace and sleeves as well as their possible effect on the adjacent joints to the knee.

## Introduction

Prophylactic knee braces (PKBs) are designed on the premise that they can protect players from sustaining debilitating injuries without inhibiting knee mobility and they have been utilized in sports without conclusive evidence regarding their efficacy in protecting the knee joint ligaments from injury [1]–[5].

However, the American Academy of Orthopaedic Surgeons position paper and several studies have illustrated that the available off-the-shelf PKBs generally provide 20–30% greater knee ligament protection [6].

With regard to the effect of PKBs on different aspects of knee function, it is claimed in some studies that knee brace use has the potential to restrict performance and may lead to early fatigue [7]–[9], while others claim that it has no negative effect on the performance [4], [10]–[12]. Hence, further research is required to alleviate potential performance concerns while using a PKB during sporting activities. The purpose of this study was to examine the effect that a PKB and two neoprene knee sleeves had upon the performance of an athlete as measured by selected laboratory parameters. The concept for manufacturing the tested PKB was to produce a low-cost PKB from components readily available in Iran as most manufactured PKBs are not available.

## Methods

### Participants

Thirty-one healthy male collegiate athletes volunteered as participants. For inclusion, players were required to be on the active college football team roster and they had no history of knee injury and knee instability which was confirmed by the university team physician. Knee instability was verified on bilateral comparison by a physical therapist who performed the Lachman, varus & valgus, McMurray, anterior/posterior drawer and Clarke's sign test. The respective physical therapist had over 11 years of clinical experience in the fields of orthopaedic and sports physical therapy. Moreover, the participants' answers to the Knee Outcome Survey-Sport Activities Scale questionnaire had to be the first choice for all of the questions indicating no symptoms and limitations in the knees [13]. The tests have been conducted in the Rehabilitation Research Center of Tehran University of Medical Sciences.

### Ethics Statement

Approval was received from the Ethics Committee of Tehran University of Medical Sciences. Prior to the start of the study, informed written consent was obtained from each subject.

### PKB/Neoprene Knee Sleeves

**Figure 1:**
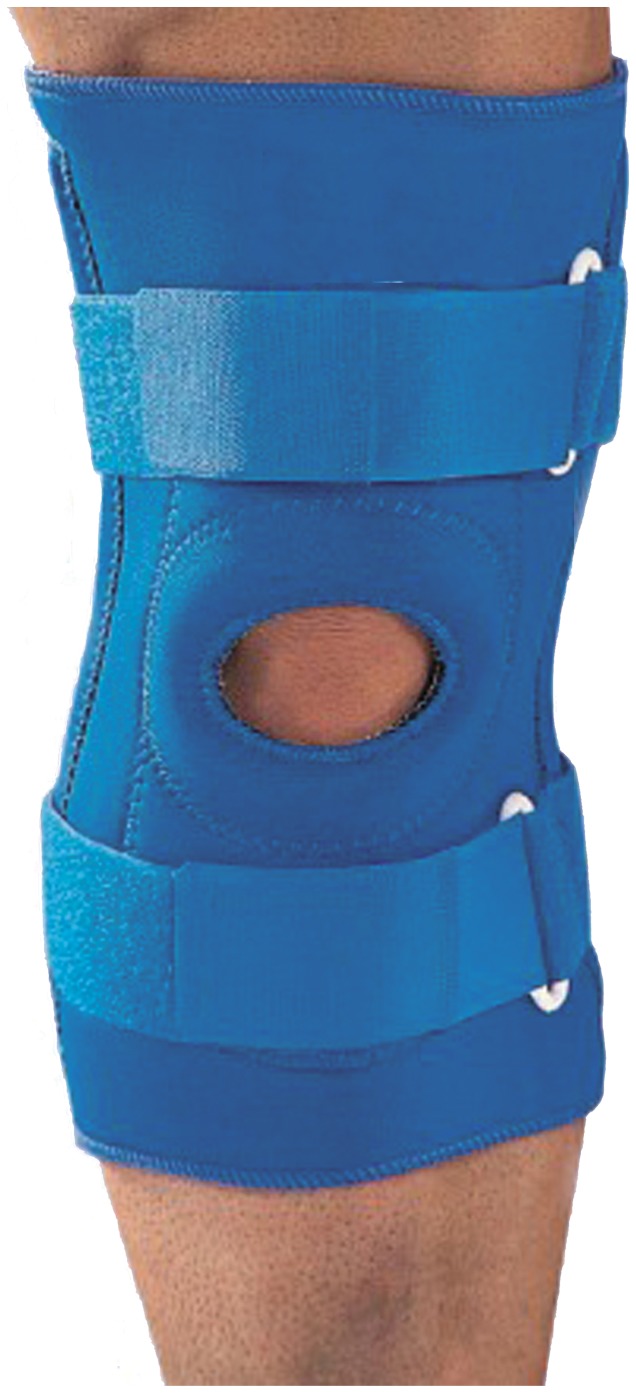
Neoprene knee sleeve with 4 metal supports.

Two neoprene knee sleeves with buttress and straps were used. One of the sleeves had four metal supports; two supporting bars on each medial and lateral side (Figure 1) and the other neoprene sleeve was with the exact design but without the metal supports.

**Figure 2:**
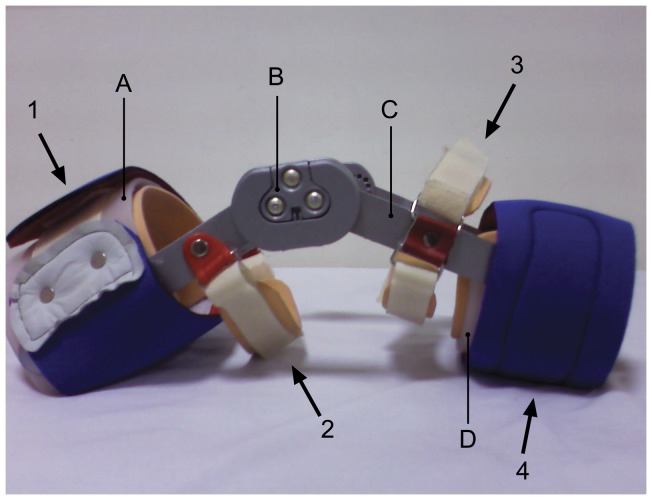
Prophylacktic knee brace.

**Figure 3:**
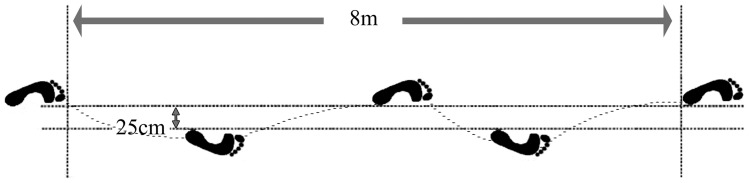
Cross-over hop test.

Moreover, a prefabricated PKB (fabricated by a certified orthotist) which was designed according to the typical designs in conventional PKBs; the design and manufacture of the PKB was supervised and verified by the faculty members of the department of Orthotics and Prosthetics of Tehran University of Medical Sciences. It included thigh and calf plastic cuffs (Figure 2; A and D), with medial and lateral aluminum uprights (Figure 2; C) and polycentric knee hinges (Figure 2; B). The hinges let full range of motion. The thigh and the calf cuff closures were made of neoprene. The stabilizing forces included a posterior force over the anterior proximal thigh (Figure 2; 1), and an anterior force on the distal posterior femur (Figure 2; 2), a posterior force at the proximal anterior tibia (Figure 2; 3), and an anterior force at the mid-shaft of the tibia (Figure 2; 4). Moreover, the lateral bars and cuffs are responsible for limiting the strains on the lateral knee ligaments by redirecting a lateral impact force away from the joint line to points more distal on the thigh and calf cuffs. A total of 6 braces have been fabricated, three for each left and right side (small, medium and large size). The weight of the medium size brace was about 0.57 kg.

### Experimental design

All tests were performed unilaterally on the braced dominant leg. The dominant leg was defined as the leg that the participants used or would use to kick a ball.

The experimental protocol consisted of isokinetic and functional tests. Subjects performed all performance tests over the four testing conditions:

1. Non-braced (NBR) or control.
2. Use of a neoprene sleeve.
3. Use of neoprene sleeve with four metal supports.
4. Use of prefabricated PKB.

The sequence of four test conditions were assigned in a random manner and participants were permitted a 10-minutes rest period between tests.

### Functional tests

Before the test, all subjects perform a pre-established warm up on a stationary bicycle for 5–8 minutes followed by 3–5 minutes of whole body stretching. After the warm up, subjects performed up to two practice tests of the two functional tests utilized for this study. Subjects first performed the cross-over hop test for distance. They performed four consecutive hops, crossing the center line with each hop as illustrated in Figure 3. No restrictions were placed on the upper extremities. Then, the single leg vertical jump (SLVJ) test began with a measure of reach height on the dominant side next to the wall with ink applied to the participants' finger. Subsequently, they performed a maximal effort single-leg jump and reached to touch the wall placing the second mark at the pick of the jump. Participants were allowed to move their arms freely and landed bilaterally, as this is a more functional movement pattern [14]. For each functional test, subjects completed three trails with a 30 seconds rest period between each trail and 5–10 minutes rest period between the two functional tests. The best result of the three trails was used for analysis [14]. This is in accordance with the procedure used in competitions and athletic performance tests which include several trials – the best trial is usually recorded as the competition or test score [15]. Validity and reliability of vertical jump [16]–[18] and cross-over hop test [19]–[22] can be found in the previous studies.

### Isokinetic tests

After the functional tests, isokinetic measurements were taken using the Biodex Multi-Joint System 3 dynamometer (Biodex Medical Systems, Inc, New York, USA) with the knee attachment. Participants were seated and secured to the apparatus with straps across the chest and thighs. The back of the seat was set at an 85° angle. The resistance pad was placed on the distal tibia. The range of motion of the knee joint was set at 0–90° [23]. Afterward, each subject performed the Biodex concentric knee extension and flexion test. The test protocol consisted of 3 repetitions at 60°/sec, 5 repetitions at 180°/sec and 10 repetitions at 300°/sec. Sixty and 180°/sec are reasonable and comfortable test velocities that seems to meet the essential requirements of testing validity and the need for information about muscle performance at the functional range. The velocity of 300°/sec was chosen as a high range to test the performance in the high velocity athletic activities. Moreover, as the higher test velocities incorporate lower reliability, more repetitions were necessary to avoid the concerns regarding the reliability of the tests. Hence, more repetitions were considered for higher test velocities in this study [23]. Previous studies have also demonstrated validity and reliability of the Biodex isokinetic dynamometer and [24], [25] a high intraclass correlation coefficients (ICC) for isokinetic testing at these three angular velocities (60, 180, 300°/sec) [25]. To authors' knowledge no other studies have tested this range of isokinetic velocities to assess PKBs, specifically the higher velocity. When each set of repetitions was completed the athletes were permitted a three minutes rest period. Finally, the isokinetic variables including peak torque to body weight ratio (PTBWR) and average power at three angular velocities of 60, 180 and 300°/sec were recorded.

### Statistical Analysis

Data was entered into the SPSS13 statistical software package (SPSS, Chicago, USA). Repeated measures analyses of variances, with the significance level of 0.05, were performed to evaluate any differences between the four test conditions. One factor of the condition of test was considered to compare the functional test results. For analyzing the isokinetic measurements two factors of condition and velocity of test was examined for the comparisons.

## Results

No statistical significance (p>0.05) was found between the four (NBR, neoprene sleeve, neoprene sleeve with four metal supports, and PKB) testing conditions while performing the SLVJ and the cross-over hop tests (Table 1). Also, there was no significant difference in PTBWR and average power over the four testing conditions (p>0.05) at any of the three angular velocities (Table 2, 3). For the isokinetic testing, there was no interaction between velocity, testing conditions, and PKB and/or neoprene sleeve use.

**Table 1:** Means of measurements of functional tests in four conditions.

	Functional tests (cm)*				
	Control	Sleeve	Sleeve with support	PKB^†^	p-value^‡^
**SLVJ** ^§^	33.2±5.6	33.45±5.7	33.0±5.6	33.23±5.2	0.537
**Hop**	771.9±95.7	778.92±85.2	782.5±90.1	770.43±93.0	0.209

* Mean ± standard deviation; ^†^Prophylactic Knee Brace; ^‡^p-values for the repeated measures analysis of variance with one factor of test conditions; ^§^Single leg vertical jump.

**Table 2:** Means of isokinetic PTBWR in three angular velocities and four testing conditions.

	PTBWR (%)*									
	Control		Sleeve		Sleeve with support		PKB^†^		p-value^‡^	
Velocity (°/sec)	Ext.^§^	Flex^≈^	Ext.	Flex.	Ext.	Flex.	Ext.	Flex.	Ext.	Flex.
**60**	281.9±50.5	120.3±23.0	273.6±52.1	118.8±24.3	286.4±42.7	118.37±23.5	280.9±54.1	116.4±24.4	0.218	0.239
**180**	204.1±28.6	107.1±16.5	205.5±27.4	106.3±16.2	210.1±28.5	108.48±15.7	202.8±31.6	104.0±17.4		
**300**	159.9±25.1	104.5±18.6	164.0±26.8	106.4±15.1	161.6±21.4	99.93±14.5	161.1±25.9	97.6±16.6		

* PTBWR, Peak Torque to Body Weight Ratio (Mean ± standard deviation); ^†^Prophylactic Knee Brace; ^‡^p-values for the repeated measures analysis of variance within the two factors of velocity and test conditions; § Extension; ^≈^Flexion.

**Table 3:** Means of isokinetic Average Power measurements in three angular velocities and four testing conditions.

	Average power (watt)*									
	Control		Sleeve		Sleeve with support		PKB^††^		p-value‡	
Velocity (°/sec)	Ext.^§^	Flex.^≈^	Ext.	Flex.	Ext.	Flex.	Ext.	Flex.	Ext.	Flex.
**60**	125.6±25.9	59.5±14.3	123.7±24.3	58.7±15.6	126.1±23.0	59.6±15.1	125.0±24.8	56.7±13.8	0.059	0.243
**180**	246.9±45.1	129.8±25.1	250.1±41.8	126.7±30.6	255.5±46.5	129.3±30.4	245.1±46.6	122.4±25.9		
**300**	273.4±49.4	142.8±28.1	288.3±55.2	142.5±26.2	277.2±52.6	138.0±33.5	274.9±57.7	139.1±30.0		

* Mean ± standard deviation; ^†^Prophylactic Knee Brace, ^‡^p-values for the repeated measures analysis of variance within the two factors of velocity and test conditions; ^§^Extension; ^≈^Flexion.

## Discussion

The results of all the isokinetic and functional tests have revealed that the PKB and neoprene knee sleeves have no negative effect on athlete performance. In comparing the NBR with the PKB and two neoprene sleeve conditions, during the SLVJ and the cross-over hop, no significant performance differences were noted. Likewise, in the isokinetic tests, wearing the PKB or sleeves did not cause a significant change in the PTBWR or the average power generated by the study participants during any of the test velocities.

There are two methods of dynamic muscle force determination that are used frequently in the lower body. One of them is isokinetic dynamometry, which gives information about the strength of specific muscles or muscle groups at a specific velocity. Since the muscular strength and power are two parameters that are recognized as influencing an athlete's performance, isokinetic torque and power were investigated in the present study as a means of understanding the effect of PKB and knee sleeves on the knee performance. The other method of determining dynamic muscular force is using a vertical jump test, which provides information about the mechanical work accomplished by the whole kinematic extensor chain. Both methods have been extensively utilized and validated in the sporting field [26]. Since the range of abnormal performance may increase with the addition of a second test [14], two functional tests were utilized in this study in conjunction with the isokinetic dynamometry.

Among the studies which assessed the effect of PKBs on the performance with similar methodologies to the present one, the results of Birmingham's study were homogeneous. Birmingham investigated the effect of the DonJoy Defiant knee brace on isokinetic peak torque at 90°/sec during knee flexion and extension in participants who had undergone ACL (anterior cruciate ligament) reconstruction, and had completed a physical therapy rehabilitation protocol. This study showed that wearing the brace decreased the flexion torque generating capacity of the knee by 7.3% which was statistically significant. However, no significant negative brace effect was exhibited for extension torques [27]. Likewise, in the present study, at the velocity of 60°/sec, PKB reduced the amount of torque generated in the flexion more than extension (3.2% and 0.4% reduction for flexion and extension respectively) comparing to the torques for the control condition, yet these reductions were not statistically significant. One of the reasons that the effect of the PKB was not significant could be the differences in the type of participants who were examined in our study and Birmingham's study (i.e. ACL-reconstructed vs. healthy). As it is shown in some studies, discrepancies in the extent to which the knee brace effects knee performance may be due to participants' current situation, including symptoms exhibited and muscle strength [4], [28]. Owing to the fact that all participants of the present study were young male athletes and that they were able to generate a mean peak torque of 282 percent of their body weight (normative measures according to Biodex ranges from 265% to 343%), it may be speculated that these participants were perfectly able to overcome the possible resistive load imposed by the PKB and neoprene sleeves. Moreover, it is possible that the potential inhibitory effect of the PKB/sleeves was not detectable given the sensitivity of the measuring devices or methods utilized in this study.

Studies by Ramsey and DeVita support the idea that supportive knee braces may evoke motor pattern changes that offer protection to ACL deficient patients [29], [30]. According to the DeVita's study, the knee joint torque variables and powers were not significantly affected by the knee brace and this coincides with the results of the current study. Furthermore, DeVita found that while wearing the functional knee brace, healthy participants were not as responsive to the brace effects as ACL-deficient patients according to the lower limb moments, especially in the knee joint [31].

Singer studied the effects of two types of knee supports on the mechanics of walking in healthy participants; a knee sleeve with bilateral hinges and a hinged-post-shell brace, and as such, this brace and knee sleeve were similar to those tested in the present study. The results revealed that flexion and extension torques of knee and other joints of the lower limb were not significantly different between brace and non-braced conditions. In addition to this, there were no statistically significant differences between the brace and sleeve [32].

Although the methods of the previous studies are unlike the current study, within all of them the effect of knee supports on two influential parameters in sports performance, i.e. muscular strength and power, were investigated [26]. Thus, as the findings of the aforementioned studies did not reveal any negative effect of knee sleeve/braces on knee torques and power in the healthy participants, it may be concluded that these results are compatible with those of the present study.

Analyzing the results of current study has shown that wearing neither the PKB nor the neoprene knee sleeves have significantly changed the isokinetic variables in any of the three fast to slow angular velocities. Again, according to functional tests there was also no significant difference between control and PKB/sleeves condition which is consistent with the results of a study by Blataci, who examined the effects of braces of similar design on vertical jump and one-leg hop test. Therefore, the results of the functional tests have supported the findings of the isokinetic tests.

In general, conventional PKBs and those that were utilized in the present study can be divided into two groups: (1) those with metal frames, and (2) knee supports with neoprene as their basic structure. The simple neoprene sleeve was utilized in the current study to understand the possible limitations that neoprene can cause when it is used as the base structure in a knee support design. In the present study, no inhibitory effect of neoprene was found; therefore, considering the positive effect of neoprene sleeves on the proprioception, utilizing them in PKBs seems quite efficient [5], [30], [33]. As a result of the fact that the PKB used in the present study had a metal frame and firm plastic shells, it may provide the knee ligaments with more support than the neoprene sleeve with four metal supports [34].

One limitation of this study was that we have only assessed the effects of bracing on the knee joint isokinetics and function. Further studies may be helpful to also investigate the interaction and compensation of adjacent joints to the knee (i.e. ankle and hip).

In this study the immediate effect of the braces were assessed; this immediate change of the knee kinetics probably predicts the subsequent changes of the knee kinetics. Thus the result of the immediate effect can be used as a guideline for indication of PKBs and knee sleeves until there will be any data regarding the long-term and on the field effects of them.

## Conclusion

The results of this study showed that the tested PKB and neoprene knee sleeves had no significant negative effect on the knee performance of male athletes, which is desirable according to the purpose of wearing theses braces; that is, these knee supports protect the knee and at the same time they do not deteriorate the performance of the athlete. In addition, no differences were observed according to whether neoprene sleeves were utilized or not; thus, coaches can choose any of these types of knee support as prophylactic or protective equipment according to the needs, type of sport, and risk of injury of the athletes.

## References

[1] Schlegel TF, Steadman JR (1997) Knee Orthoses for Sports-Related Disorders. In: Goldberg B, editor. Atlas of Orthoses and Assistive Devices. 3rd ed. Philadelphia: Mosby. pp.420–421.

[2] Najibi S, Albright JP (2005) The use of knee braces, part 1: Prophylactic knee braces in contact sports. Am J Sports Med 33: 602–611. 15788733 10.1177/0363546505275128

[3] Pietrosimone BG, Grindstaff TL, Linens SW, Uczekaj E, Hertel J (2008) A systematic review of prophylactic braces in the prevention of knee ligament injuries in collegiate football players. J Athl Train 43: 409–415. 18668174 10.4085/1062-6050-43.4.409 PMC2474821

[4] Sforzo GA, Chen NM, Gold CA, Frye PA (1989) The effect of prophylactic knee bracing on performance. Med Sci Sports Exerc 21: 254–257. 2733572

[5] Baltaci G, Aktas G, Camci E, Oksuz S, Yildiz S, et al (2011) The effect of prophylactic knee bracing on performance: balance, proprioception, coordination, and muscular power. Knee Surg Sports Traumatol Arthrosc 19: 1722–1728. 21468615 10.1007/s00167-011-1491-3

[6] Rishiraj N, Taunton JE, Lloyd-Smith R, Woollard R, Regan W, et al (2009) The potential role of prophylactic/functional knee bracing in preventing knee ligament injury. Sports Med 39: 937–960. 19827861 10.2165/11317790-000000000-00000

[7] Styf JR, Nakhostine M, Gershuni DH (1992) Functional knee braces increase intramuscular pressures in the anterior compartment of the leg. Am J Sports Med 20: 46–49. 1554073 10.1177/036354659202000112

[8] Borsa PA, Lephart SM, Fu FH (1993) Muscular and functional performance characteristics of individuals wearing prophylactic knee braces. J Athl Train 28: 336–344. 16558250 PMC1317738

[9] Hansen (1985) The Preventive Use of the Anderson Knee Stabilizer in Football. Physician & Sportmedicine 14: 257–260.

[10] Greene DL, Hamson KR, Bay RC, Bryce CD (2000) Effects of protective knee bracing on speed and agility. Am J Sports Med 28: 453–459. 10921634 10.1177/03635465000280040301

[11] Liggett CL, Tandy RD, Young JC (1995) The effects of prophylactic knee bracing on running gait. J Athl Train 30: 159–161. 16558328 PMC1317850

[12] Tegner Y, Pettersson G, Lysholm J, Gillquist J (1988) The effect of derotation braces on knee motion. Acta Orthop Scand 59: 284–287. 3381659 10.3109/17453678809149364

[13] Irrgang J, Safran M, Fu F (1996) The Knee Ligamentous and Meniscal Injuries. In: Zachazewski J, Magee D, Quillen W, editors. Athletic Injuries and Rehabilitation. Philadelphia: W.B. Saunders Co. pp.623–692.

[14] Zachazewski JE, Magee DJ (1996) Return to Competition: Functional Rehabilitation. In: Zachazewski JE, Magee DJ, Quillen WS, editors. Athletic Injuries and Rehabilitation. Philadelphia: W.B. Saunders pp.245–252.

[15] Aragón LF (2000) Evaluation of Four Vertical Jump Tests: Methodology, Reliability, Validity, and Accuracy. Meas Phys Educ Exerc Sci 4: 215–228.

[16] McElveen MT, Riemann BL, Davies GJ (2010) Bilateral comparison of propulsion mechanics during single-leg vertical jumping. J Strength Cond Res 24: 375. 20072063 10.1519/JSC.0b013e3181c06e0b

[17] Moir G, Shastri P, Connaboy C (2008) Intersession reliability of vertical jump height in women and men. J Strength Cond Res 22: 1779–1784. 18824929 10.1519/JSC.0b013e318185f0df

[18] Cordova ML, Armstrong CW (1996) Reliability of ground reaction forces during a vertical jump: Implications for functional strength assessment. J Athl Train 31: 342–345. 16558421 PMC1318919

[19] Noyes FR, Barber SD, Mangine RE (1991) Abnormal lower limb symmetry determined by function hop tests after anterior cruciate ligament rupture. Am J Sports Med 19: 513–518. 1962720 10.1177/036354659101900518

[20] Clark NC, Gumbrell CJ, Rana S, Traole CM, Morrissey MC (2002) Intratester reliability and measurement error of the adapted crossover hop for distance. Phys Ther Sport 3: 143–151.

[21] Clark NC (2001) Functional performance testing following knee ligament injury. Phys Ther Sport 2: 91–105.

[22] Bolgla L, Keskula D (1997) Reliability of lower extremity functional performance tests. J Orthop Sports Phys Ther 26: 138. 9276854 10.2519/jospt.1997.26.3.138

[23] Devir Z (2004) Isokinetics of the Knee Muscles. In: Devir Z, editor. Isokinetics: Muscle Testing, Interpretation and Clinical Applications. 2nd ed. Edinburgh: Churchill Livingstone. pp.150.

[24] Drouin JM, Valovich-mcLeod TC, Shultz SJ, Gansneder BM, Perrin DH (2004) Reliability and validity of the Biodex system 3 pro isokinetic dynamometer velocity, torque and position measurements. Eur J Appl Physiol 91: 22–29. 14508689 10.1007/s00421-003-0933-0

[25] Feiring D, Ellenbecker T, Derscheid G (1990) Test-retest reliability of the Biodex isokinetic dynamometer. J Orthop Sports Phys Ther 11: 298. 18796902 10.2519/jospt.1990.11.7.298

[26] Kovaleski JE, Heitman RJ (2000) Testing and Training the Lower Limb Extremity. In: Brown LE, editor. Isokinetics in Human Performance. first ed. Champaign: Human Kinetics. pp.179.

[27] Birmingham TB, Kramer JF, Kirkley A (2002) Effect of a functional knee brace on knee flexion and extension strength after anterior cruciate ligament reconstruction. Arch Phys Med Rehabil 83: 1472–1475. 12370890 10.1053/apmr.2002.35093

[28] Lu TW, Lin HC, Hsu HC (2006) Influence of functional bracing on the kinetics of anterior cruciate ligament-injured knees during level walking. Clin Biomech (Bristol, Avon) 21: 517–524. 10.1016/j.clinbiomech.2005.12.017 16494979

[29] DeVita P, Lassiter T Jr, Hortobagyi T, Torry M (1998) Functional knee brace effects during walking in patients with anterior cruciate ligament reconstruction. Am J Sports Med 26: 778–784. 9850778 10.1177/03635465980260060701

[30] Ramsey DK, Wretenberg PF, Lamontagne M, Nemeth G (2003) Electromyographic and biomechanic analysis of anterior cruciate ligament deficiency and functional knee bracing. Clin Biomech (Bristol, Avon) 18: 28–34. 10.1016/s0268-0033(02)00138-9 12527244

[31] DeVita P, Torry M, Glover KL, Speroni DL (1996) A functional knee brace alters joint torque and power patterns during walking and running. J Biomech 29: 583–588. 8707784 10.1016/0021-9290(95)00115-8

[32] Singer JC, Lamontagne M (2008) The effect of functional knee brace design and hinge misalignment on lower limb joint mechanics. Clin Biomech (Bristol, Avon) 23: 52–59. 10.1016/j.clinbiomech.2007.08.013 17920738

[33] Theoret D, Lamontagne M (2006) Study on three-dimensional kinematics and electromyography of ACL deficient knee participants wearing a functional knee brace during running. Knee Surg Sports Traumatol Arthrosc 14: 555–563. 16598506 10.1007/s00167-006-0072-3

[34] France EP, Paulos LE, Jayaraman G, Rosenberg TD (1987) The biomechanics of lateral knee bracing. Part II: Impact response of the braced knee. Am J Sports Med 15: 430–438. 3674266 10.1177/036354658701500502

